# Treatment outcomes of adult patients with recurrent tuberculosis in relation to HIV status in Zimbabwe: a retrospective record review

**DOI:** 10.1186/1471-2458-12-124

**Published:** 2012-02-13

**Authors:** Kudakwashe C Takarinda, Anthony D Harries, Satyanarayana Srinath, Tsitsi Mutasa-Apollo, Charles Sandy, Owen Mugurungi

**Affiliations:** 1 AIDS & TB Unit, Ministry of Health & Child Welfare, 2nd Floor, Mkwati Building, Corner Livingstone Avenue and Fifth Street, Harare, Zimbabwe; 2 International Union Against Tuberculosis and Lung Disease, 68 Boulevard Saint-Michel, 75006 Paris, France; 3 Department of Clinical Research, London School of Hygiene and Tropical Medicine, Keppel Street, London WCE1E 7HT, UK; 4 International Union Against Tuberculosis and Lung Disease, South-East Asian Union Office, C-6, Qutub Institutional Area, New Delhi, India

**Keywords:** HIV, Recurrent tuberculosis, Treatment outcomes, Zimbabwe

## Abstract

**Background:**

Zimbabwe is a Southern African country with a high HIV-TB burden and is ranked 19^th ^among the 22 Tuberculosis high burden countries worldwide. Recurrent TB is an important problem for TB control, yet there is limited information about treatment outcomes in relation to HIV status. This study was therefore conducted in Chitungwiza, a high density dormitory town outside the capital city, to determine in adults registered with recurrent TB how treatment outcomes were affected by type of recurrence and HIV status.

**Methods:**

Data were abstracted from the Chitungwiza district TB register for all 225 adult TB patients who had previously been on anti-TB treatment and who were registered as recurrent TB from January to December 2009. The Chi-square and Fischer's exact tests were used to establish associations between categorical variables. Multivariate relative risks for associations between the various TB treatment outcomes and HIV status, type of recurrent TB, sex and age were calculated using Poisson regression with robust error variance.

**Results:**

Of 225 registered TB patients with recurrent TB, 159 (71%) were HIV tested, 135 (85%) were HIV-positive and 20 (15%) were known to be on antiretroviral treatment (ART). More females were HIV-tested (75/90, 83%) compared with males (84/135, 62%). There were 103 (46%) with relapse TB, 32 (14%) with treatment after default, and 90 (40%) with "retreatment other" TB. There was one failure patient. HIV-testing and HIV-positivity were similar between patients with different types of TB. Overall, treatment success was 73% with transfer-outs at 14% being the most common adverse outcome. TB treatment outcomes did not differ by HIV status. However those with relapse TB had better treatment success compared to "retreatment other" TB patients, (adjusted RR 0.81; 95% CI 0.68 - 0.97, *p *= 0.02).

**Conclusions:**

No differences in treatment outcomes by HIV status were established in patients with recurrent TB. Important lessons from this study include increasing HIV testing uptake, a better understanding of what constitutes "retreatment other" TB, improved follow-up of true outcomes in patients who transfer-out and better recording practices related to HIV care and treatment especially for ART.

## Background

The global burden of tuberculosis (TB) is highest in the Sub-Saharan Africa region [1] and this can be attributed to the high prevalence of human immunodeficiency virus infection (HIV) which is known to increase the risk of developing TB [2,3]. Tuberculosis cases are categorised as new and recurrent, the latter referring to patients who have been previously treated. Recurrence of TB can be attributed to relapse with a persistent *Mycobacterium tuberculosis *strain [4] or reinfection with a different strain [5,6]. Several studies have shown that HIV infection increases the rate of TB recurrence after successful completion of TB treatment in high HIV endemic areas, with recurrence often being due to a reinfection [6-9].

Despite evidence from clinical trial settings indicating that rates of recurrent TB can be reduced by administration of rifampicin-based treatment [10], there has been a steady increase in the number of tuberculosis (TB) case notifications that are categorised as recurrent or "retreatment". Since 1995, when the global DOTS framework was launched, up to 2008, the annual number of retreatment cases globally has increased from 59,240 to 775,403 [1]. In the WHO Africa region, the annual number of notified retreatment TB cases had increased from 15,133 in 1995 to 135,564 by the end of 2008, with 53,190 being relapse cases and 82,374 being registered in other retreatment categories [1]. In general, the treatment outcomes of retreatment cases are not as good as with new cases of TB as shown by global statistics for the 2008 cohorts, whereby only 72% of retreatment cases (and 71% from the Africa region) successfully completed treatment [1].

Zimbabwe is a country in Southern Africa with a population of 13 million and a large HIV-TB burden. Zimbabwe, which ranks 19^th ^among the 22 Tuberculosis high burden countries, had a TB incidence rate of 762 cases per 100,000 population and an HIV co-infection prevalence of above 75% in 2008 [1]. There were 3631 (10%) notified retreatment cases out of a total of 36,650 cases registered in 2008, and these cases had a treatment success rate that was reported at 73% compared to 69% and 74% for new smear-positive TB cases and new-smear negative/extra-pulmonary TB cases respectively [1].

In 2008, the estimated number of retreatment TB cases with multi-drug resistant (MDR) TB (resistant to both isoniazid and rifampicin) was 8.3% (95% CI; 2.9-22). However, from 2005 to 2009, there were no notified retreatment cases that were actually tested for drug resistance [1], so this estimate cannot be confirmed. HIV-infected TB patients should now receive a package of cotrimoxazole preventive therapy (CPT) and antiretroviral therapy (ART), both of which can reduce case fatality and increase treatment success if administered early enough during anti-TB treatment [11-15]. It is therefore important that TB patients should undergo HIV testing as soon as TB is diagnosed, so that they can access these adjunctive therapies.

Given the high HIV prevalence in Zimbabwe and the growing importance of recurrent TB, we determined in one district in Zimbabwe (i) the type of tuberculosis in adult patients registered with recurrent TB, ii) the proportion that were HIV tested with their results and (iii) the treatment outcomes of recurrent TB patients in relation to type of TB and HIV status.

## Methods

### Study design

This was a retrospective record review of adult patients registered as "recurrent TB".

### Study setting

All public health facilities in Chitungwiza were selected for this study. Chitungwiza is a high density dormitory town which is situated about 20 km from Zimbabwe's capital city, Harare, and has a population of 343 147 [16]. All health facilities consisting of a government central hospital and four polyclinics, which are under the Chitungwiza City Health Department, offer general health services including TB treatment services. However, TB laboratory diagnosis through direct smear microscopy examination is only carried out at the government central hospital where all collected sputum smears in the district are sent. All health facilities offer HIV counselling and testing services and cotrimoxazole prophylaxis free of charge for confirmed HIV-positive patients as recommended by the Zimbabwe National TB Control Programme (NTP). In 2009 ART initiation and HIV treatment and care services in Chitungwiza were only offered at an ART initiating facility at the government central hospital where all HIV-positive patients in the district were referred.

#### TB Diagnosis and Definitions

Patients with recurrent TB will have been previously treated for TB and are classified according to standard WHO case definitions as either a relapse case (a patient who completed treatment and presents again with smear-positive PTB), treatment after failure (a patient who failed first line treatment), treatment after default (a patient who defaulted from treatment and presents again with smear-positive PTB) or "retreatment other" (all other recurrent TB cases) [2]. In the Zimbabwe NTP, TB is diagnosed based on an initial clinical diagnosis and then a confirmative laboratory diagnosis through direct smear microscopy of sputum smears [17]. Those with smears positive for acid-fast bacilli are diagnosed as smear-positive PTB, whilst those with negative smears are referred for a chest radiograph. If the latter is suggestive of TB, the patient is diagnosed as smear-negative PTB. Extra-pulmonary TB (EPTB) is mainly diagnosed on clinical grounds, along with circumstantial evidence and supporting specific diagnostic tests [17].

After TB diagnosis, health workers at each health facility complete notification forms and patient treatment cards for all recurrent TB patients indicating diagnosis, patient category, treatment regimen and monitoring indices. Patient information is then entered into the DOTS register at each health facility, which is then later compiled into the District TB register for Chitungwiza. During the period of the study, HIV testing was routinely offered to all TB patients, using the parallel testing algorithm [18]. However as from 2010 the serial testing algorithm was adopted.

After diagnosis and registration, all recurrent TB patients are given an 8-month standard re-treatment regimen with first line drugs (2SHRZE/1HRZE/5HRE) regardless of HIV status [17]. In Chitungwiza, treatment was administered daily through health facility based DOT during the first 2 months of the intensive phase. Thereafter patients were given anti-TB drugs that ranged from weekly to monthly supplies until completion of the 8-month treatment period. Patients have their sputum specimens reviewed at 3 months, 5 months and at the end of treatment as recommended in the national guidelines [17]. In Zimbabwe, cotrimoxazole preventive therapy (CPT) is recommended for the whole duration of TB treatment [19] whilst ART is recommended between 2 and 8 weeks after commencing TB treatment [20]. National TB treatment outcomes are in line with WHO guidelines and are classified as treatment success (cured plus treatment completed), defaulted, died, transferred out or treatment failure. Cohort analyses of the registered TB patients are done to declare their TB treatment outcomes 12 months after the start of TB treatment.

### Data collection

Data were collected for all adult TB patients registered in the District TB register for Chitungwiza between 1 January 2009 and 31 December 2009 who had previously been on TB treatment for more than one month. An adult was defined as a person 18 years and older. Patient data were abstracted from the District TB registers between October 2010 and February 2011 using a data collection form, and variables that were collected included:- TB registration number, age, sex, type and category of recurrent TB, HIV status (HIV test done or not done, HIV-negative or HIV-positive), treatment outcome (treatment success, died, defaulter, transferred out and failure) and cotrimoxazole use. For patients registered in 2009, ART data were not available in the district TB registers, hence information about ART use in HIV-infected TB patients was obtained from the ART register at Chitungwiza Central hospital ART clinic were HIV-infected patients were referred for ART initiation in this district. TB registration numbers were collected as patient identifiers but however patient names had to be used to trace HIV-infected TB patients commenced on ART from the ART register.

### Statistical analysis

Patient information was coded, entered and cleaned using Epidata version 3.1 statistical software. Data were then imported and analysed using Stata 10 (Stata Corporation, College Station, Texas). The Chi-squared test or alternatively the Fischer's Exact test was used to determine if there were associations between categorical variables. TB treatment outcomes were categorised into binary data with possible outcome events being treatment success, death, transfer-outs, defaulters and treatment failure. Poisson regression with error variance was then used to estimate multivariate-adjusted relative risks for associations between the various TB treatment outcomes and HIV status, type of recurrent TB, sex and age.

The modified Poisson regression model with the robust error variance option was used as it is a better approach for estimating relative risks for cohort studies compared to using logistic regression [21]. Assumptions of this statistical model were not violated as the model provides relative biases and percentages of confidence interval coverage which are reliable with a minimum sample size of 100 [21]. Relative risks for treatment failure, however, could not be calculated as there were no data in each subgroup to perform meaningful Poisson regression analysis. The 5% significance level was used in this study.

### Ethics

Access to data in the TB registers was granted by the Ministry of Health and Child Welfare whilst ethics approval was obtained from the Medical Research Council of Zimbabwe and the Union Ethics Advisory Group in Paris, France.

## Results

### Characteristics of patients with recurrent TB

There were 225 registered patients with recurrent TB, of whom 135 (60%) were males. Results of HIV testing and documented referral of HIV-infected TB patients to ART are shown in Additional file 1: Figure S1. Just over 70% of patients were tested for HIV and of these patients 85% were HIV-positive, but only 15% were documented as being initiated on ART. There were no data in the registers about initiation of HIV-infected TB patients on CPT. Table 1 shows results of HIV testing and HIV serostatus in relation to type of TB, sex and age. Proportions of patients HIV tested were similar across all the different types of recurrent TB (*p *= 0.476). However, HIV-positivity had a borderline association with type of TB (*p *= 0.052) being highest among relapse cases (89%) compared to treatment after default patients (70%) - *p *= 0.037. Though more females were tested (75/90, 83%) compared to males (84/135, 62%) - *p *= 0.001, HIV-positivity was similar between the 2 groups. HIV testing was highest in the 45 to 54 year age group (87%) compared to other age groups but HIV-positivity was similar (*p *> 0.99) across the age groups.

**Table 1:** HIV testing and HIV serostatus in relation to TB type, sex and age

Variable	Number registered	Number (%) HIV tested	^ǂ^P-value	Number (%) HIV-positive	P-value
All patients	225	159 (71)	-	135 (85)	-
Type of TB*					
Relapse	103	72 (70)	0.476	64 (89)	0.052
Treatment after default	32	20 (63)		14 (70)	
Retreatment other	90	67 (74)		57 (85)	
**Sex**					
Female	90	75 (83)	0.001	66 (88)	0.357
Male	135	84 (62)		69 (82)	
**Age group**					
< 25	16	11 (69)	0.039	10 (91)	> 0.99
25 - 44	147	99 (67)		83 (84)	
45 - 54	39	34 (87)		29 (85)	
≥ 55	23	15 (65)		13 (87)	

*TB *tuberculosis, *HIV *human immunodeficiency syndrome

### TB treatment outcomes in relation to type of TB and HIV status

Treatment outcomes for all patients with recurrent TB in relation to HIV status, type of TB, sex and age group are shown in Table 2. The treatment success rate was 73% for all TB patients, regardless of HIV status. Patients who transferred out (14%) constituted the majority of those with adverse treatment outcomes, with 15 (49%) being HIV-positive with undocumented ART status. For all patients, treatment success rates were similar regardless of HIV status (HIV test done or not done, HIV test positive or negative). Treatment success rates were also similar when stratified by sex and age group, with transfer outs being the most common adverse outcomes. TB treatment success rate was however highest in those with relapse TB (80%) compared to "retreatment other" TB patients (adjusted RR 0.81; 95% CI 0.68 - 0.97, *p *= 0.02). For all other adverse treatment outcomes, there were no significant multivariate-adjusted relative risks for associations with HIV status, type of TB, sex and age.

**Table 2:** TB treatment outcomes for all recurrent TB patients in relation to HIV status, type of TB, sex and age

Variable	Number registered	Treatment Outcomes ^b^				
		
		Treatment success n (%)	Death n (%)	Defaulted n (%)	Transfer out n (%)	Treatment failure n (%)
**All patients**	225	163 (73)	13 (6)	14 (6)	32 (14)	1 (< 1)
**HIV status^a^**						
HIV-negative	21	15 (71)	2 (10)	2 (10)	2 (10)	0 (0)
HIV-not done	64	44 (69)	2 (3)	5 ( 8)	13 (20)	0 (0)
HIV-positive (ART not documented)	115	87 (76)	6 (5)	5 ( 4)	15 (13)	1 (1)
HIV-positive (on ART)	20	14 (70)	2 (10)	2 (10)	1 ( 5)	0 (0)
**Type of TB**						
Relapse TB	103	82 (80)	4 (4)	6 (6)	11 (11)	0 (0)
Treatment after default	32	21 (68)	2 (6)	3 (10)	5 (16)	0 (0)
Retreatment other	90	60 (67)*	7 (8)	5 (6)	16 (18)	1 (1)
**Sex**						
Female	90	68 (76)	5 (6)	6 (7)	10 (11)	0 (0)
Male	135	95 (71)	8 (6)	8 (6)	22 (16)	1 (1)
**Age group (in years)**						
< 25	16	12 (80)	1 (7)	0 (0)	2 (13)	0 (0)
25 - 44	147	108 (74)	6 (4)	11 (8)	21 (14)	0 (0)
45 - 54	39	26 (67)	5 (13)	2 (5)	5 (13)	1 (3)
≥ 55	23	17 (74)	1 (4)	1 (4)	4 (17)	0 (0)

^a ^2 patients with unrecorded HIV status and 3 with HIV-indeterminate results have been excluded; ^b ^2 patients with unrecorded TB treatment outcomes have been excluded

*TB *tuberculosis, *ART *antiretroviral treatment, *HIV *human immunodeficiency virus

Significant statistical comparisons using multivariate Poisson regression: * *p *< 0.05 compared with relapse TB

## Discussion

This study in Chitungwiza district in Zimbabwe showed a very high HIV prevalence in patients registered with recurrent TB, but we did not find significant differences in treatment outcomes in relation to HIV status, age or sex. However, relapse TB patients had better treatment success compared to "retreatment other" TB patients.

There are a number of important findings from our study. Although HIV testing should be offered to all registered patients with TB, nearly one third of those with recurrent TB had no HIV test done. We do not know the reasons for this, and this particular issue deserves further investigation. HIV testing uptake was much better in females than males, and better strategies of encouraging males to be HIV tested need to be found and assessed.

The majority of patients with recurrent TB were HIV-positive, yet only 15% were documented to be receiving ART. There may be several reasons for this that include poor referral and access to ART services by HIV-TB patients in Chitungwiza district or a poor recording and reporting system. For patients registered in 2009, the only way to find whether HIV-TB patients had accessed ART was to review the ART registers for Chitungwiza district and try to match the names of HIV-infected TB patients with those from the TB register - matching was not possible and this is therefore a limitation of the study. HIV-TB patients who had transferred out and started ART in another district would also not be identified by this method and therefore missed for the purposes of this study. Since 2010, HIV and TB activities have been integrated into TB registers, and there should now be better data on ART. There was also no record of CPT use in the TB register and this needs improvement as well.

Finally, there was a high transfer out rate which adversely impacted on treatment success. A transfer out is defined as a patient who has been transferred to a health facility in another district and the treatment outcome results have not been reported back to the referring treatment unit who have the duty to report on these patients. This is an area where the Zimbabwe NTP needs to improve and ensure that centres who receive transfer-in patients report back the treatment outcomes to the facilities that initiated TB treatment.

This was a district based study where data were collected for TB patients under routine programme conditions. Hence, it may be difficult to ensure completeness, consistency and accuracy of data. Our study sample was small therefore it may have reduced the power of the study. We also were unable to adjust for the potential confounding effect of ART and TB treatment compliance and disease severity when assessing the association between TB treatment outcomes and HIV status as the data were unavailable.

## Conclusions

In conclusion, there are some important lessons to learn from the study. More attention should be paid to dissecting out the various categories of patients who are classified together under "retreatment other" to determine whether outcomes are similar or different. Relapse patients appear to do better, but the power of our sample may have been insufficient to detect this. Improving the follow up of outcomes in transferred out patients coupled with better recording practices of HIV care and treatment will allow better analysis of the relationship between TB treatment outcomes and HIV status. Transfer outs may also be lessened by integration of TB treatment services and ART initiation in all health facilities.

There needs to be a higher uptake of HIV testing in TB patients, especially in males. If those found HIV-positive are followed up and given CPT and early ART, this may improve overall treatment outcomes. There needs to be much better recording practices of HIV care and treatment, especially with regard to CPT and ART. Future research of a prospective nature with a larger cohort of recurrent TB patients may more clearly establish the impact of HIV testing on TB treatment outcomes under programmatic conditions.

## Competing interests

The authors declare that they have no competing interests.

## Authors' contributions

KT designed the study, collected and analysed data, wrote the first draft and coordinated the writing of the subsequent drafts and the final paper. ADH, SS, TMA, CS and OM contributed to the design of the study and all subsequent drafts of the paper. All authors read and approved the final paper.

## Pre-publication history

The pre-publication history for this paper can be accessed here:

http://www.biomedcentral.com/1471-2458/12/124/prepub

## Supplementary Material

Additional file 1 **Figure S1**. HIV testing status and known referral to antiretroviral treatment for recurrent tuberculosis patients in Chitungwiza district, Zimbabwe (Jan - Dec 2009). Click here for file
