# Supplementary figures and images for: Treatment outcomes of adult patients with recurrent tuberculosis in relation to HIV status in Zimbabwe: a retrospective record review
Source: BMC Public Health. 2012 Feb 13;12:124. doi: 10.1186/1471-2458-12-124 (PMC3305664; doi:10.1186/1471-2458-12-124)

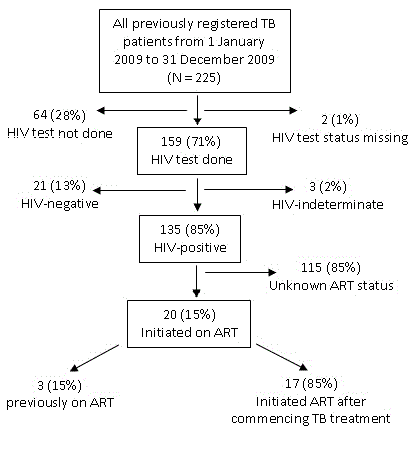

Supplement: Additional file 1 — Figure S1. HIV testing status and known referral to antiretroviral treatment for recurrent tuberculosis patients in Chitungwiza district, Zimbabwe (Jan - Dec 2009). [file 1471-2458-12-124-S1.PNG]
